# Case Report: Propranolol Therapy for Infantile Tremor Syndrome in a Child With Vitamin B12 Deficiency

**DOI:** 10.3389/fped.2021.774747

**Published:** 2021-11-24

**Authors:** Amélie Cyr, Ryan Frehlick, David Stammers, Megan Crone

**Affiliations:** ^1^Department of Pediatrics, College of Medicine, University of Saskatchewan, Saskatoon, SK, Canada; ^2^College of Medicine, University of Saskatchewan, Saskatoon, SK, Canada; ^3^Division of Pediatric Hematology and Oncology, Jim Pattison Children's Hospital, Saskatoon, SK, Canada; ^4^Division of Pediatric Neurology, Jim Pattison Children's Hospital, Saskatoon, SK, Canada

**Keywords:** neurology, hematology, vitamin B12 deficiency, infantile tremor syndrome, propranolol

## Abstract

Vitamin B12 deficiency in childhood presents with a wide variety of symptoms including anemia, failure to thrive and developmental delays. It is a diagnostic consideration in children who are exclusively breastfed or have minimal solid intake, especially if their mother is vegetarian or has underlying vitamin B12 deficiency. Infantile tremor syndrome (ITS) has been associated with vitamin B12 deficiency. ITS presents with neurological symptoms such as developmental delays and tremors. The tremors seen in ITS can be profound and interfere with daily functioning. Different therapies have been tried for those tremors without much evidence or information regarding their efficacy and dosing regimens. We present the case of a 13-month-old girl with vitamin B12 deficiency who developed ITS with significant tremors after initiation of vitamin B12 therapy. She was treated with propranolol which resulted in significant improvement in her tremors. This case highlights the efficacy and safety of propranolol for the treatment of ITS in the context of vitamin B12 deficiency.

## Introduction

Children with vitamin B12 deficiency can present with anemia, pancytopenia, failure to thrive, hypotonia and developmental delays and/or regression (1–3). Vitamin B12 deficiency can be seen in infants who are exclusively breastfed by mothers who are vegetarian or who are themselves vitamin B12 deficient (1–3). Investigations in children with vitamin B12 deficiency can reveal macrocytic anemia, leukopenia, thrombocytopenia, high serum homocysteine levels and high lactate dehydrogenase (LDH) levels (4). Peripheral blood smears may show macrocytic hypochromic oval erythrocytes, anisocytosis, poikilocytosis, and hypersegmentation of neutrophils (5). Furthermore, head imaging may reveal cerebral atrophy and delayed myelination patterns for age (1). Early recognition and treatment of vitamin B12 deficiency can lead to significant improvement in terms of neurodevelopment and prevent long term neurological consequences (1–3).

Infantile tremor syndrome (ITS) has been associated with vitamin B12 deficiency. ITS is defined as the “tetrad of pallor, developmental delay/regression, skin pigmentation, and brown scanty scalp hair” (6). Tremors may occur later in the course of ITS and tend to be jerky in character. Tremors usually involve facial muscles and infants can also present with “tremulous vocalization or cry” (6). It has been documented that tremors may be triggered by vitamin B12 replacement in children who are deficient (6). The etiology and pathophysiology of ITS are still unknown, but several studies have linked ITS to vitamin B12 deficiency (6). Tremors seen in ITS have been treated with different modalities such as propranolol, sedatives and anticonvulsants, but no study has looked at their efficacy and safety profile (6).

## Case Report

A previously healthy 13-month-old girl was referred to a tertiary pediatric care center for further investigations and management of her pancytopenia. She was initially seen by a general pediatrician in the community for concerns of a 2-month history of tiredness, decreased appetite and failure to thrive. A complete blood count (CBC) was done after noticing her pallor which revealed pancytopenia. Her hemoglobin level was 52 (range 105–145) g/L, her platelet count was 120 (range 150–400) × 10^9^/L and her leukocyte count was 6.6 (range 7–20) × 10^9^/L. Her physical examination was significant for pallor, tachycardia, hypotonia, and sparse scalp hair. Her weight, length and head circumference were below the third percentile for gender and age. Upon review, she was also noted to have gross motor delays: she still needed assistance to sit or stand and she was unable to crawl or walk. In terms of diet, she was mostly breastfed. Limited solids were introduced at 6 months of life, but she then developed oral aversion secondary to episodes of constipation. The family history was unremarkable except for her mother having iron deficiency anemia and hypothyroidism.

**Figure 1:**
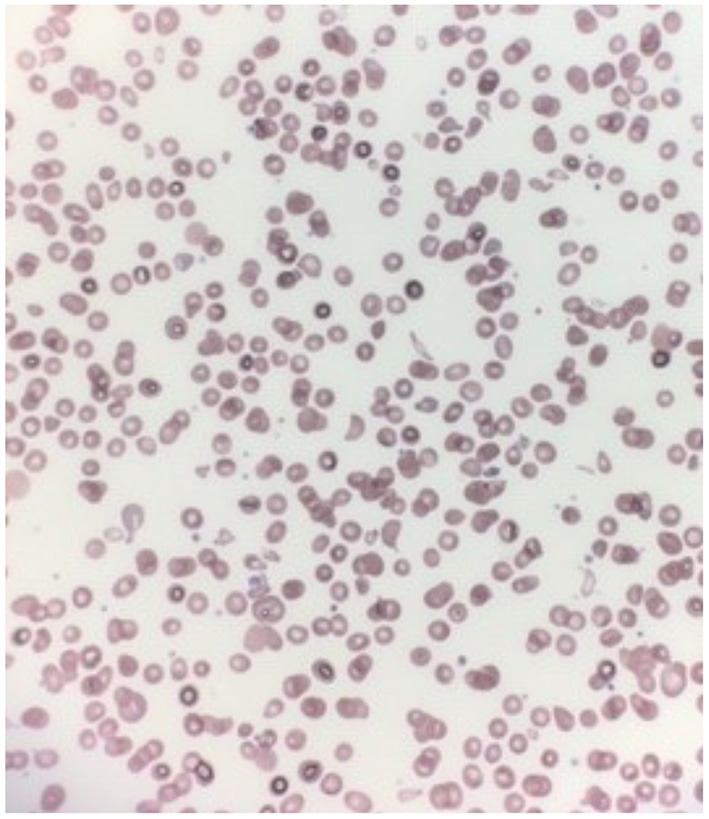
Marked poikilocytosis on patient's peripheral blood smear.

Further investigations revealed that her anemia was macrocytic: her MCV was 107.4 fL (range 79–97 fL). She also had undetectable vitamin B12 levels (<100 pg/mL, normal range 160–950 pg/mL), as well as high lactate dehydrogenase (LDH) levels (>4,300 U/L, normal range 373–618 U/L). Her reticulocyte count was also inappropriately within the normal range (61 × 10^3^/μL, normal range 19–73 × 10^3^/μL) given her significant anemia. Her other nutrients including iron and folic acid were within normal range. Immunoglobulin levels were not measured. Her peripheral blood smear showed marked poikilocytosis as shown in Figure 1. Her work-up for malignancy including a bone marrow aspirate was negative. Her work-up for other causes of bone marrow suppression was also negative (negative Hepatitis, Parvovirus B19 and Epstein-Barr Virus panels). Given these findings, her pancytopenia and developmental delays were thought to be secondary to severe vitamin B12 deficiency. Unfortunately, testing for serum homocysteine and urine methylmalonic acid was only done after starting treatment and results were normal. Considering that her oral intake was from breastmilk exclusively, her mother's vitamin B12 levels were measured, and she was found to be vitamin B12 deficient as well (undetectable vitamin B12 levels < 100 pg/mL). Her mother was referred for further evaluation of the underlying cause of her vitamin B12 deficiency. Pediatric gastroenterology was also involved to assess for potential gastrointestinal causes of vitamin B12 deficiency including H. pylori gastritis, small intestine inflammation and pancreatic insufficiency. These investigations came back normal, including a negative H. pylori stool antigen testing, normal stool alpha-1-antitrypsin levels (195 mg/dL, normal range 95–200 mg/dL), a negative celiac disease screen, a negative intrinsic factor blocking antibody testing and normal stool calprotectin levels (62 ug/g). Based on these findings and her exclusive breastfeeding, the most likely cause of her vitamin B12 deficiency was thought to be secondary to dietary deficiency knowing that her mother was vitamin B12 deficient as well. The patient was started on intramuscular vitamin B12 injections at 1,000 mcg daily.

**Figure 2:**
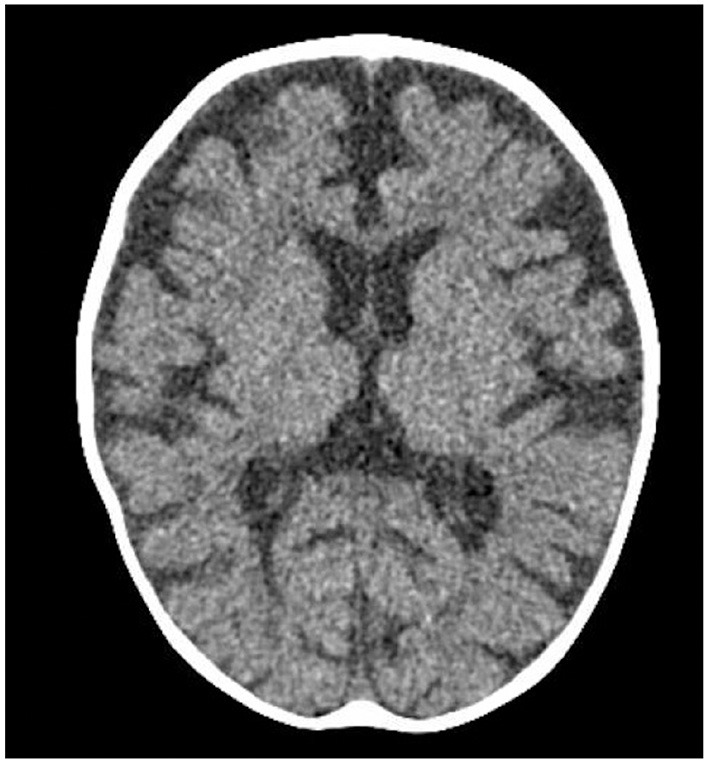
Axial head CT image showing marked cerebral atrophy with reduced white matter volume.

**Figure 3:**
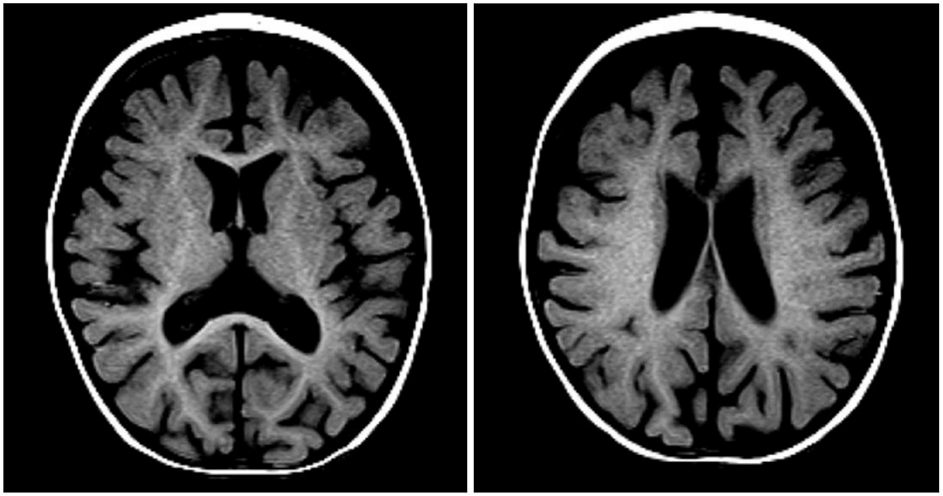
Axial T1 MRI images showing reduced white matter volume resulting in enlargement of the CSF spaces with age-appropriate myelination pattern.

Two days after starting her vitamin B12 therapy, she was noted to have tremors. The tremors involved her arms and legs and occasionally her lips and tongue. The tremors were high amplitude, low frequency jerks that were suppressible. They resembled shaking movements aggravated by action. The tremors were also present during sleep but at a lesser intensity. She did not show other movement abnormalities. There were no vital signs changes associated with these episodes and her pupils were always reactive. Occasionally, her eyes were wandering during the tremor episodes. Lorazepam was trialed for possible seizures, but it did not have any effect. Given her risk of sinus venous thrombosis related to her profound anemia and dehydration upon presentation, a head computed tomography venogram (CTV) scan was done to rule-out any intracranial anomaly. No acute intracranial pathology was identified but significant atrophy with decreased white matter volume was seen (Figure 2). The tremors were initially intermittent but could last up to 5 h. At that time, the pediatric neurology team became involved in the care of this patient. An electroencephalogram (EEG) was done to rule-out seizure activity and was normal. A magnetic resonance imaging (MRI) of her head was completed and the findings were consistent with the head CT results. Her MRI showed reduced white matter volume resulting in enlargement of the CSF spaces with age-appropriate myelination pattern (Figure 3). There was no cerebrospinal fluid analysis done. She was diagnosed with infantile tremor syndrome (ITS), which was likely triggered by her vitamin B12 treatment, as described in the literature (6). Her daily tremors, accompanied with bleating sounds, worsened at bedtime and were starting to impact her ability to feed herself. A decision to treat was made given the functional impairment. Given the lack of robust evidence in terms of therapy and dosing, propranolol was chosen considering its good safety profile in infants with hemangiomas and some reports of efficacy in tremors associated with ITS (2). A starting dose of 0.5 mg/kg/day divided twice daily was used, which is a safe, standard starting dose used in infantile hemangiomas. There was a plan for further titration to 1 mg/kg/day based on her response, however, after 1 day of treatment, she was already showing improvement in her tremors. Within a week of treatment, her tremors had decreased in frequency and intensity and were no longer interfering with her functioning. Her blood glucose levels, heart rates and blood pressures were closely monitored during the initiation of propranolol and she did not experience any side effects from this therapy.

The patient's reticulocyte count and vitamin B12 levels showed great response to the vitamin B12 therapy. Within 5 days of treatment, her reticulocyte count increased to 436 × 10^3^/μL and her vitamin B12 levels were >2,000 pg/mL. From a dietary perspective, she was started on nutritional supplementation *via* a nasogastric tube. Speech language pathology was also involved to help manage her oral aversion and to slowly introduce solids to her diet. The patient improved dramatically throughout her stay on the pediatric ward. After receiving blood transfusions and vitamin B12 injections, her energy had improved. She made great progress from an oral aversion perspective and by the end of her stay, some solids were reintroduced to her diet. She restarted gaining weight while on nasogastric feeds. She was also showing some progress in terms of gross motor development with better central tone and head control. At the time of discharge, she still had mild tremors that were not interfering with her functioning and she was continued on the same dose of propranolol.

She was closely followed by general pediatrics, hematology and neurology as an outpatient. She continued to show dramatic improvements in terms of neurodevelopment: within a month, she was sitting unsupported, was able to stand with minimal support and started to crawl. Her oral intake also increased significantly, and she was transitioned off of nasogastric tubes 4 months after her admission. Given the significant improvement in her tremors, she was weaned off of the propranolol after 6 weeks of treatment without any rebound in her symptoms.

## Discussion

Vitamin B12 deficiency can present with a wide variety of symptoms including developmental delay and regression. Vitamin B12 deficiency should be considered in infants mostly or exclusively breastfed, especially if their mother is vegetarian, has a diet poor in meat or has an underlying pernicious anemia (6). Many cases were previously reported in developing countries, but it is a diagnosis to consider in any setting, especially given the increasing popularity of vegetarianism and veganism. In fact, some studies have shown that the prevalence of these diets has increased substantially in the past decade and can be estimated to about 2–5% in North Americans (7). This case, along with many others reported in the literature, stresses the importance of early detection and treatment of vitamin B12 deficiency in infancy to improve neurodevelopmental outcomes and avoid permanent long-term neurological consequences (8).

Infantile tremor syndrome has been associated with many cases of vitamin B12 deficiency and there is growing evidence that this nutritional deficiency could actually be the cause of ITS (5). Of note, tremors can also be seen in other nutritional deficiencies such as zinc and magnesium (6). Zanus et al. reviewed cases of ITS related to vitamin B12 deficiency and common findings to our case include progressively increasing shaking movements usually starting 2-8 days after onset of vitamin B12 replacement (9).

Different treatment options have been tried to address the tremors associated with ITS such as carbamazepine (10, 11), sedatives like phenobarbital (9), and propranolol (2). However there is little information regarding the dosing regimens used and associated outcomes. Propranolol was selected in this case given its efficacy reported in some case series of ITS (2) and its relatively safe side effect profile compared to sedatives and anticonvulsants trialed in the literature. As there is little reported guidance, the propranolol dosing regimen in this case along with a monitoring protocol was based on the one used for infantile hemangiomas at our pediatric center. The patient experienced significant improvement in her tremors without any adverse reaction.

This case highlights the efficacy and safety of propranolol for the treatment of ITS in the context of vitamin B12 deficiency. From a parental perspective, addressing these tremors provided reassurance and allowed their daughter to progress in terms of developmental skills. However, it is a limited report with only one case. Further studies are needed to identify the optimal treatment protocol for tremors associated with infantile tremor syndrome and vitamin B12 deficiency.

## Data Availability Statement

The original contributions presented in the study are included in the article/supplementary material, further inquiries can be directed to the corresponding author/s.

## Ethics Statement

Written informed consent was obtained from the minor(s)' legal guardian/next of kin, for the publication of any potentially identifiable images or data included in this article.

## Author Contributions

AC, RF, DS, and MC conceptualized and designed the study, drafted the initial manuscript, and reviewed and revised the manuscript. All authors approved the final manuscript as submitted and agree to be accountable for all aspects of the work.

## Conflict of Interest

The authors declare that the research was conducted in the absence of any commercial or financial relationships that could be construed as a potential conflict of interest.

## Publisher's Note

All claims expressed in this article are solely those of the authors and do not necessarily represent those of their affiliated organizations, or those of the publisher, the editors and the reviewers. Any product that may be evaluated in this article, or claim that may be made by its manufacturer, is not guaranteed or endorsed by the publisher.

## Abbreviations

ITS: Infantile tremor syndrome.

## References

[1] Bousselamti A El Hasbaoui B Echahdi H Krouile Y. Psychomotor regression due to vitamin B12 deficiency. Pan Afr Med J. (2018) 30:152. 10.11604/pamj.2018.30.152.12046 30374398 PMC6201603

[2] Gowda VK Kolli V Benakappa A Srinivasan VM Shivappa SK Benakappa N. Case series of infantile tremor syndrome in tertiary care Paediatric Centre from Southern India. J Trop Pediatr. (2018) 64:284–8. 10.1093/tropej/fmx062 28977620

[3] Roumeliotis N Dix D Lipson A. Vitamin B12 deficiency in infants secondary to maternal causes. CMAJ. (2012) 184:1593–8. 10.1503/cmaj.112170 22711730 PMC3470622

[4] Aslinia F Mazza JJ Yale SH. Megaloblastic anemia and other causes of macrocytosis. Clin Med Res. (2006) 4:236–41. 10.3121/cmr.4.3.236 16988104 PMC1570488

[5] Means R Fairfield K. Clinical Manifestations and Diagnosis of Vitamin B12 and Folate Deficiency. (2021). Available online at: https://www.uptodate.com/contents/clinical-manifestations-and-diagnosis-of-vitamin-b12-and-folate-deficiency (accessed July 9th, 2021).

[6] Goraya JS Kaur S. Infantile tremor syndrome: a review and critical appraisal of its etiology. J Pediatr Neurosci. (2016) 11:298–304. 10.4103/1817-1745.199475 28217150 PMC5314841

[7] Sakkas H Bozidis P Touzios C Kolios D Athanasiou G Athanasopoulou E. Nutritional status and the influence of the vegan diet on the gut microbiota and human health. Medicina. (2020) 56:88. 10.3390/medicina56020088 32098430 PMC7073751

[8] Mathey C Di Marco JN Poujol A Cournelle MA Brevaut V Livet MO. Stagnation pondérale et régression psychomotrice révélant une carence en vitamine B12 chez 3 nourrissons. Underweight and psychomotor regression revealing vitamin B12 deficiency in 3 infants. Arch Pediatr. (2007) 14:467–71. 10.1016/j.arcped.2007.01.014 17412572

[9] Zanus C Alberini E Costa P Colonna F Zennaro F Carrozzi M. Involuntary movements after correction of vitamin B12 deficiency: a video-case report. Epileptic Disord. (2012) 14:174–80. 10.1684/epd.2012.0507 22591802

[10] Tunçer GO Köker A Köker SA Aba A Tural Kara T Coban Y. Infantile tremor syndrome after peroral and intramuscular vitamin B12 therapy: two cases. Zwei Fälle Klin Padiatr. (2019) 231:274–7. 10.1055/a-0981-6355 31408906

[11] Gupta R Rawat AK Singh P Gupta J Pathak A. Infantile tremor syndrome: current perspectives. Res Rep Trop Med. (2019) 10:103–8. 10.2147/RRTM.S180604 31308787 PMC6615725

